# QRS-T angle as a predictor of pulmonary arterial hypertension: A review

**DOI:** 10.1097/MD.0000000000032320

**Published:** 2023-01-13

**Authors:** Bo Li, Xuhan Liu, Baoguo Wang, Xiuqing Liu, Weihua Zhang

**Affiliations:** a Department of Cardiovascular Medicine, The First Hospital of Jilin University, Changchun, China.

**Keywords:** ECG, noninvasive techniques, pulmonary hypertension, QRS-T angle, vectorcardiography

## Abstract

Spatial QRS-T angle is a vector projection of ventricular gradient, reflecting the heterogeneity of ventricular repolarization. The QRS vector is projected into three dimensional (3D) space to obtain the frontal QRS_f_ vector and Tf vector. The frontal QRS-T Angle can be obtained by simple calculation. In several studies of diseases that cause pulmonary hypertension, there has been evidence that changes in the QRS-T Angle can predict disease. Electrocardiogram (ECG) is a cheap and noninvasive test, and its clinical significance in the diagnosis of pulmonary hypertension should be further studied.

## 1. Introduction

Wilson proposed in 1934 that the spatial QRS-T Angle is a vector projection of the ventricular gradient. It reflects the representation of repolarization and depolarization in three-dimensional (3D) space and is a measure of the duration and amplitude of ventricular heterogeneous action potentials and the heterogeneity of ventricular repolarization. In the depolarized state, depolarization occurs from the endocardium to the epicardium, whereas in the repolarized state, repolarization occurs from the epicardium to the endocardium. Repolarization begins when the inner membrane of the heart is exposed to high pressure. This delays the recovery of endocardial action potentials, producing a time difference between epicardial repolarization and endocardial repolarization, the ventricular gradient,^[1]^, which will alter the spatial QRS-T Angle. Ventricular gradients are typical of healthy myocardium. The QRS-T spatial Angle was more associated with pulmonary hypertension than with right atrial (RA) or ventricular dilatation and/or hypertrophy. This may be because structural or functional changes in dilation or hypertrophy alone are not sufficient to account for the entire myocardial ventricular gradient.

The frontal QRS_f_ vector and T_f_ vector are obtained by projecting the QRS vector in 3D space. The Angle formed between the frontal QRS_f_ and T_f_ wave vectors is called the frontal QRS-T Angle. Frontal QRS-T Angle (f [QRS-T]) corresponds to the Angle between the QRS axis of the depolarization indicator and the T-wave axis of the repolarization indicator of the electrocardiogram (ECG). The F (QRS-T) value on the 12-lead ECG recording can be easily calculated and can be calculated by removing the T-wave axis from the QRS axis recorded by the automatic reporting^[2]^ ECG device.

Independent of the underlying etiology, PH leads to RV pressure overload and dysfunction, which can be detected with echocardiography.^[3,4]^ Echocardiography is also a valuable technique for diagnosing PH, particularly with respect to PH associated with left heart disease or chronic heart disease. Yet, echocardiography alone is insufficient to confirm a diagnosis of PH, which requires right heart catheterization. Also echocardiography has a large number of false positive results. An increase in systolic blood pressure is not always associated with an increase in pulmonary vascular resistance. However, the combination of echocardiography and QRS-T Angle can rule out pulmonary hypertension as a suspected disease. Henkens et al found that rats with RVH experienced a drastic increase in QRS/T angle by 25 days with the terminal repolarization sequence changing from concordant to reverse sequence. As an adaptive mechanism, these changes were considered by these authors. Additionally, RV load- (stretch-) dependent voltage-gated potassium channels are likely to be downregulated.^[5,6]^ This is a possible physiological reason for the change of QRS-T angle in pulmonary hypertension.

### 1.1. QRS-T angle

The QRS-T angle is derived from ECG analysis. It was first proposed in 1934, and since then it has been widely studied because of its particularity in the study of ventricular gradient. Ventricular gradient is the vector sum of spatial QRS and T-angle.^[2]^ Spatial QRS-T angles can also be considered “secondary T waves” and occur when abnormal depolarization causes abnormal repolarization, including changes in ventricular conduction (including ventricular premature beats, ventricular pacing, and bundle branch block), without morphological heterogeneity of primary action potentials. The spatial QRS-T Angle needs to be calculated by complex formulas, while the frontal QRS-T Angle is easier to calculate and statistically study. In the field of pulmonary hypertension, there is no clear evidence of a significant difference between the 2 calculation methods.

Frontal QRS_f_ and T_f_ vectors are generated by 3D QRS vector projection. The Angle formed by frontal QRS-T vector and frontal QRS-T vector is called frontal QRS-T Angle respectively. Using a standard 12-lead ECG, the frontal QRS-T Angle can be easily calculated by calculating the absolute value of the difference between the frontal QRS axis and the T-axis. If this difference exceeds 180 degrees, the frontal QRS-T Angle is calculated as 360 degrees minus the absolute difference between the frontal QRS axis and the t-axis. Multiple studies have examined overall mortality in patients with abnormally wide QRS-T angles, all of which have been positive. Patients with baseline cardiovascular disease had a nearly 3-fold increased risk of all-cause death compared with those with normal QRS-T angles.^[7–12]^ Despite the mild nature of this relationship, it was significant in individuals without baseline cardiovascular disease, with a hazard ratio of 1.28:1 for those with wide-space QRS-T angles.

QRS-T Angle is not only a noninvasive test, but also a promising research direction for its predictive effect on PAH. The application of QRS-T Angle in PAH is described in detail in this paper.

### 1.2. QRS-T angle and pulmonary hypertension

#### 1.2..1. Pulmonary hypertension caused by acute pulmonary embolism.

At present, there is no specific diagnosis of acute pulmonary embolism ECG results. In addition, the study showed that no specific ECG findings determined the efficacy of thrombolytic therapy and predicted clinical outcome in the group receiving thrombolytic therapy. Ecg recordings can reflect the changes in the axis of the heart caused by right ventricular (RV) enlargement and the changes in the hyperpolarization-depolarization process during hypoxia. Pulmonary embolism aggravates RV load, thereby affecting frontal QRS-T Angle.^[13]^ The changes of QRS-tangle in frontal region after thrombolytic therapy in patients with pulmonary embolism may be related to the prognosis of thrombolytic therapy.^[13]^

#### 1.2..2. Pulmonary hypertension due to atrial septal defect (ASD).

Amr El-Bokl first proposed the idea of using spatial QRS-T Angle to distinguish pulmonary hypertension with secondary ASD from ASD without pulmonary hypertension.^[14]^ After research, they believed that Using a spatial QRS-T angle can differentiate secundum ASD patients with pulmonary hypertension from ASD patients without pulmonary Hypertension. And in their study, it was the only derived ECG variable that reliably predicted pulmonary hypertension.

#### 1.2..3. Idiopathic pulmonary arterial hypertension (IPH) and chronic thromboembolic pulmonary hypertension (CTEPH) cause pulmonary hypertension.

In patients with IPH and CTEPH,^[15]^ the change of QRS-T Angle is strongly correlated with SPAP, RV size, RA size, and RV systolic and diastolic function. An analysis has shown that QRS-T Angle can be combined with changes in echocardiography^[15]^ for the definitive diagnosis of IPH and CTEPH patients.

#### 1.2..4. Chronic obstructive pulmonary disease.

Chronic obstructive pulmonary disease is often characterized by structural and hemodynamic changes in the heart, and because these changes typically develop late, it is often difficult to diagnose by symptoms and signs alone. Therefore, simpler and practical noninvasive diagnostic tests are necessary in order to diagnose the disease as early as possible and improve the prognosis. Through experimental research, the data finally drawn is by increasing QRS-T By 10°, 0.8 mm Hg increase in pulmonary artery systolic pressure was observed.^[16]^

## 2. Prospect and conclusion

Pulmonary hypertension is a serious and highly fatal disease. Combined with other noninvasive screening tools, including echocardiography, QRS-T analysis can be helpful in early diagnosis of pulmonary hypertension. Its high negative predictive value makes it a potentially powerful early screening method for pulmonary hypertension. Spatial QRS-T Angle is currently an effective tool for screening pulmonary hypertension in children and adolescents.^[1]^ However, no study has demonstrated the difference between spatial QRS-T and frontal QRS-T Angle in the diagnosis of pulmonary hypertension. ECG as a cheap noninvasive auxiliary examination method should be further explored in the early diagnosis of pulmonary hypertension.

## Author contributions

**Conceptualization:** Bo Li.

**Formal analysis:** Baoguo Wang.

**Funding acquisition:** Weihua Zhang.

**Project administration:** Xiuqing Liu.

**Supervision:** Xuhan Liu, Baoguo Wang, Weihua Zhang.

**Validation:** Baoguo Wang, Xiuqing Liu.

**Writing – original draft:** Bo Li, Xuhan Liu.
